# Predicting transient performance of a heavy-duty gaseous-fuelled engine using combined phenomenological and machine learning models

**DOI:** 10.1177/14680874241305732

**Published:** 2024-12-29

**Authors:** Navid Balazadeh, Sandeep Munshi, Mahdi Shahbakhti, Gordon McTaggart-Cowan

**Affiliations:** 1 School of Sustainable Energy Engineering, Simon Fraser University, Surrey, BC, Canada; 2 Westport Fuel Systems Inc, Vancouver, BC, Canada; 3 Mechanical Engineering Department, University of Alberta, Edmonton, AB, Canada

**Keywords:** Natural gas engine, direct injection, heavy-duty class 8 trucks, 1-D phenomenological model, fuel consumption, greenhouse gas emissions, machine learning

## Abstract

Decarbonizing long-haul goods transportation poses a substantial challenge. High-efficiency natural gas (NG) engines, which retain the efficiency of a diesel engine but reduce the carbon content of the fuel, offer substantial potential for near-term greenhouse gas (GHG) reductions. A fast-running model that can predict engine performance, GHG and air pollutant emissions is critical to assessing this approach for different applications and vehicle drivetrain configurations. This paper presents the development, validation and application of an engine system model that adapts GT-SUITE™’s phenomenological DI-Pulse predictive model to predict the performance and emissions of a 6-cylinder NG engine using a high pressure direct-injection combustion process. The model includes the engine air exchange system, enabling the prediction of the engine and in-cylinder conditions and overall performance over transient drive cycles. The engine model with a fixed set of calibration parameters captures the complex high-pressure direct injection combustion process and generates time-resolved parameters that are fed into a coupled machine learning model to predict emissions, including nitrogen oxide (NOx) and methane (CH_4_) emissions. While the 1-D model’s predictions for CH_4_ were not accurate, coupling the 1-D engine model with a machine learning model has been shown to substantially improve the estimation of CH_4_ emissions and allow accurate prediction of engine total GHG emissions over different duty cycles. The model has been validated using transient engine dynamometer data and is then applied to assess performance and emissions over several regulatory and real-world long-haul drive cycles. The model showed an average error of less than 5% in steady operation. Cumulative errors of NOx and CH_4_ emissions in studied cycles were also less than 10%. The results showed that CH_4_ share in total GHG emissions ranges from 0.2% to 1.4% over various drive cycles. By predicting engine performance and emissions, the developed combined model has considerable potential for use in engine evaluation studies, especially when combined with new technologies across different duty cycles.

## Introduction

Heavy-duty commercial vehicles are critical to economic activity. Propelled by diesel engines, they emit significant amounts of greenhouse gases (GHGs) and local air pollutants such as nitrogen oxides (NOx). Over the past decades, distances covered and payloads carried by long-haul heavy-duty vehicles (HDVs) have risen dramatically, resulting in substantial increases in GHGs despite improvements in engine, powertrain, and vehicle system efficiencies.^1^ Class 8 vehicles, which have a gross vehicle weight rating (GVWR) of 33,000 pounds or more, make up only 9% of the HDV fleet in North America, yet they emit nearly half of the NOx and GHGs.^2^ As a result, regulatory agencies in North America, Europe and elsewhere are imposing ever more stringent regulations on both NOx emissions and GHGs from commercial vehicles.^3–6^ Low-carbon fuels such as methane and renewable natural gas offer the potential for substantial reductions in net GHG emissions when used in a high efficiency engine and integrated with advanced powertrain systems. To quantify the potential benefits of such combinations, it is critical to have a fast-running simulation that can predict engine performance, fuel consumption (FC) and emissions over a wide range of transient duty cycles that are representative of real-world uses.

Combining improvements in engine, powertrain, and vehicle technologies has led to substantial reductions in on-road fuel consumption and corresponding reductions in tailpipe GHG emissions. The peak brake thermal efficiency (BTE) in modern diesel engines used in on-road HDVs is approaching 50% through a combination of improved combustion and incremental reductions in friction and engine parasitic losses.^7,8^ Waste-heat recovery strategies, through turbo-compound or organic Rankine cycle systems, have been demonstrated to reach 54% peak BTE.^9^ However, the potential GHG reductions from these approaches are limited by the relatively high carbon:energy ratio of the diesel fuel. Combining a high efficiency engine, powertrain, and vehicle with a lower-carbon fuel, such as natural gas (NG), offers a short-term route to reduce the GHG emissions from HDVs. To realize this potential, it is critical that the NG fuelled engine retains the efficiency of the diesel engine. A route to achieving this is to retain the diesel engine’s predominantly mixing-controlled combustion process, but to replace the liquid diesel fuel injection with directly injected natural gas.^9,10^

Directly injected natural gas when the piston is near the top-dead-centre and ignited by a diesel pilot injected prior to the natural gas can retain diesel-like efficiency. NG high-pressure direct-injection (HPDI) engine, which uses a small amount of diesel for ignition (typically less than 5% across much of the engine map), is as efficient as diesel engines while emitting 15%–25% lower GHGs.^11,12^ The pilot and main gas injections can be controlled independently using a dual concentric needle injector. Many of the advances in diesel engine technology can be carried over to a low-carbon gaseous fuelled engine using this approach.^11^

The injection and combustion processes of a natural gas HPDI engine have been studied extensively using optical techniques,^13–16^ engine performance studies,^12,17,18^ and computational modelling.^19–22^ The compression process is identical to an equivalent diesel engine, with an injection of a small amount of diesel fuel as the piston approaches top-dead-centre. The auto-ignition of the diesel pilot increases in-cylinder temperature and provides multiple ignition sources for the NG ignition. The natural gas injection is initiated at around the same time as the diesel pilot ignites. The injected NG ignites in the hot zones of pilot combustion products, and the NG jet pushes the pilot zone to the piston wall zone; however, some unburned NG can penetrate past the pilot reaction zones and mix with air.^13,23^ This overlean mixture is thought to be one of the main mechanisms of unburned CH_4_ emissions in the HPDI engine.^13^ After ignition, directly injected NG faces different combustion stages and primarily burns in non-premixed (diffusion) mode. A high heat release rate (HRR) for NG combustion is attributed to a partially premixed phase. In the subsequent non-premixed phase, the reaction zone progresses toward the injector, forming a quasi-steady lifted jet flame. In the last combustion phase, the remaining oxidation products react with the oxidizer throughout the combustion chamber.^13,23^

Although NG emits less CO_2_ than diesel during combustion, significant CH_4_ emissions can lower or even negate the GHG benefits of NG fuel,^24^ highlighting an accurate prediction of this emission. For the HPDI engine, different studies employed Computational Fluid Dynamics (CFD) models to predict engine’s emissions and performance. The studies show that CFD models have good accuracy in reproducing experimental results; for NOx emissions, errors are below 5%, while for CH_4_, carbon monoxide (CO) and particulate matter (PM) emissions, errors could increase to 10%–20%.^14,19,21^ Most studies have utilized these models to inspect the effect of different pilot and main injection strategies^21^ or other engine parameters, such as compression ratios (CR) and excess air ratios (EAR),^19^ on engine efficiency and emissions. Huang et al.^22^ could accurately predict engine-out emissions, including CH_4_ emissions, using large-eddy simulation with an error of 10%–12%. Their results show that engine low-load operation increased CH_4_ emissions due to low local temperature and slow flame propagation. While comprehensive 3-D simulation models, grounded in physical principles, are capable of accurately replicating physical phenomena, they have a high computational cost and are impractical in engine system-level evaluations compared to fast-running 1-D simulation models.

Appropriate prediction of the net GHG benefits in the HPDI engine depends on an accurate predictive engine model applicable to different real-world driving cycles. A 1-D simulation enables system-level evaluation of the engine, and unlike CFD models, they can be implemented in transient conditions. However, the lack of a spatial resolution for temperature or concentration fields in 1-D models could result in less accurate predictions of unburned fuel and byproduct emissions from partial combustion. To resolve this limitation, the 1-D model can be improved using alternative methods, such as machine learning (ML) models.

Grey-box models, which combine ML and physics-based methods, are particularly appealing as they combine the benefits of physics-based and ML models.^25^ Several studies have focused on predicting emissions for diesel engines in real-world conditions using ML models, such as neural network multi-layer perceptron (MLP),^26–28^ Support Vector Regressor (SVR)^29–31^ and Gaussian process regression (GPR).^32^ However, the implementation of such models in real-world cycles is rare for NG-DI engines. This study aims to bridge this gap by integrating the 1-D model with an ML model. Coupling the ML and engine models, where the physically representative verified engine model provides input parameters for the ML emissions model, offers combined performance and emissions prediction capability over a range of conditions.

This work has demonstrated the development of a phenomenological combustion model at steady-state modes. Since the map-based engine data misses the transient effects,^33^ the next objective is to develop a transient engine model. Finally, as the current sub-model within the 1-D model is primarily for diesel engines, improvements are needed to better predict transient CH_4_ emissions for the HPDI engine. For this reason, the final objective is to integrate the transient model with an ML model to enhance CH_4_ predictions for real-world and regulatory cycles.

## Methodology

In this study, the engine analyzed is a heavy-duty late-cycle direct-injection natural gas engine utilizing pilot ignition. It features a fuel system that employs Westport Fuel Systems (WFS) HPDI dual concentric needle injectors. The specifications of the engine are detailed in Table 1. The engine model is developed in GT-SUITE™ v. 2022. The engine model development included a predictive phenomenological combustion model calibrated for the power cylinder, followed by the development of a multi-cylinder engine that was validated under both steady-state and transient operating modes. Validation was conducted using data provided by WFS for a range of steady-state points and transient cycles. These results were collected using engine dynamometer tests at the WFS engine research facility in Vancouver, BC, Canada. The engine was installed on a test bed with a 500 kW AVL Schneider AC dynamometer capable of steady-state and transient operation over the entire engine operating map. The test facilities included charge air conditioning to maintain stable intake air temperature and pressure. A Horiba MEXA-7500DEGR motor exhaust gas analyzer was used to measure engine-out gaseous species emissions. The gas analyzer uses a flame ionization detector (FID) to measure total hydrocarbons (THC) with its built-in non-methane hydrocarbon (NMHC)-cutter to measure methane emissions. The gas analyzer also uses a chemiluminescence detector (CLD) to measure NOx emissions. The in-cylinder pressure was measured using flush-mounted transducers with data collected at 0.1 °CA resolution for a minimum of 100 cycles. Air flow was measured using a Meriam laminar flow element in the intake air stream, diesel fuel flow was measured using an AVL733 microbalance, and gas flow was measured using a Micromotion Coriolis flow meter.

**Table 1.** Engine specifications.

Parameter	Description
Engine configuration	Inline six-cylinder
Power per cylinder, kW	57
Max. brake mean effective pressure (BMEP), bar	23
Fuel system	Direct injection, dual concentric needle HPDI
Aspiration	Fixed-geometry, wastegate turbocharger
Displacement per cylinder, L	2.1
Compression ratio	17:1
Bore × stroke, mm × mm	131 × 158

### Validation data

**Figure 1.**
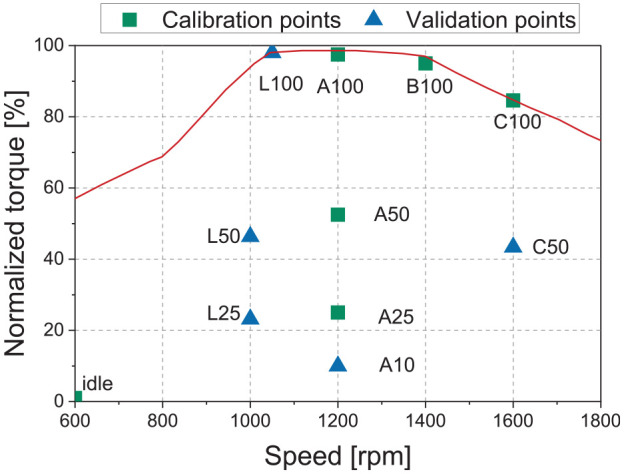
Engine steady-state operating points. Note that the *y*-axis is normalized with regard to the maximum value.

The industrial project partner collected the engine data on a multi-cylinder research engine. Eleven steady-state operating points were collected and are shown in Figure 1. Six representative points that are particularly important for the in-use operation were selected to develop the model. Since the utilized model in this study is a predictive model, which means that it can predict burn rates and operate beyond calibration points, additional operating points are needed to verify the model’s accuracy. For this reason, a further five points were selected to establish the model’s ability to operate outside of the range of values where it was calibrated.

**Figure 2.**
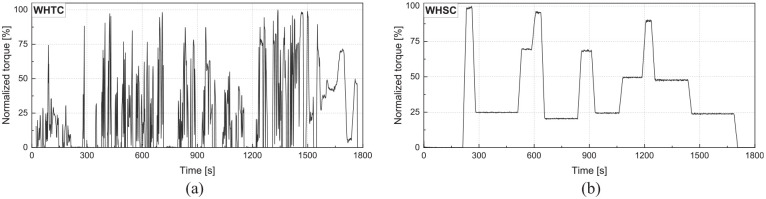
Transient torque data for (a) WHTC and (b) WHSC cycles.

Transient data was collected at a sample interval of 0.1 s in both the World Harmonized Transient Cycle (WHTC) and the World Harmonized Stationary Cycle (WHSC). The data comprised intake and exhaust pressure, temperature, intake air flow measurements, and fuel flow (NG and diesel). The demand torque data for these transient cycles are shown in Figure 2. An AVL emissions bench was used to measure the undiluted engine-out emissions before the after-treatment system. The collected data was used to develop and validate the engine 1-D model and machine learning models.

### Phenomenological combustion model

To model the entire engine system over transient cycles, it is critical to use a fast-running combustion model that can offer an adequate prediction of the actual physical combustion process. GT-SUITE™ contains several physical and chemical sub-models used to generate the physical model of the engine and calibrate the combustion model. The DI-Pulse predictive model is used to model the HPDI combustion. However, this model is generally used for direct injection diesel engines with single to multi-pulse injections. To accommodate HPDI combustion, we configured two injectors in the GT-SUITE™ model as a new approach.

The phenomenological combustion model can predict the combustion rate and the associated emissions by discretizing the engine into three zones: the main unburned zone, the spray unburned zone (fuel and entrained gas), and the burned zone (combustion products). In addition, DI-Pulse can predict in-cylinder conditions, such as pressure, heat release rate, and temperature; however, this model is not spatially representative.^34^ The combustion model can evaluate the interactions between pulses and include sub-models of relevant physical processes. Four main sub-models in the DI-Pulse model contain the related calibration parameters. The sub-models include entrainment, ignition, and premixed and non-premixed combustion.^34^ The entrainment sub-model handles the intermixing of different pulses. Since the original ignition sub-model of DI-Pulse is for diesel fuel, the ignition delay multiplier accounts for the HPDI ignition delay for its diesel pilot spray. After the pulse ignition and partially premixed combustion phase, the remaining unmixed fuel and entrained gas can burn in the diffusion combustion phase, in which the rate is controlled by the diffusion combustion rate multiplier.

### Engine steady-state model

**Figure 3.**
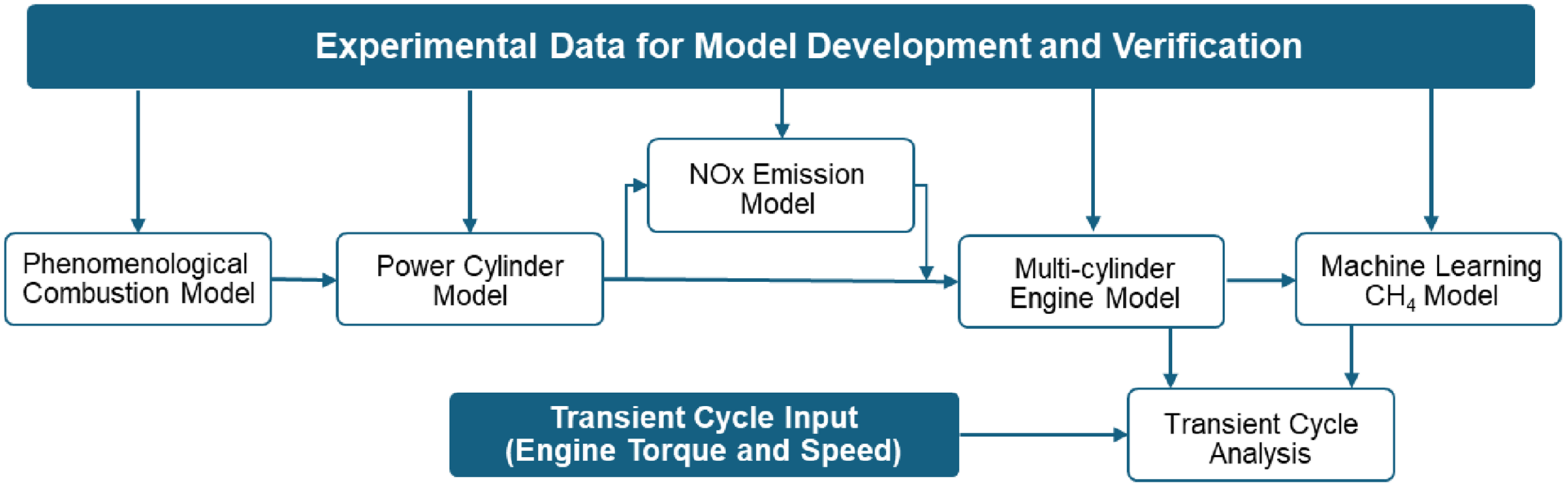
Overall structure, development processes and their interconnections to achieve the final model.

The different steps are taken in developing the 1-D model, mainly the closed-cycle analysis and combustion calibration using a genetic algorithm (GA).^35^ While the development of the steady-state combustion model (power cylinder model) is a critical piece, the overall process involves multiple stages, each contributing to the creation of the final transient model. The overall structure and interconnection between these different stages are summarized in Figure 3, providing an overview of how individual components integrate to achieve the final transient model.

A critical preliminary step in developing a representative engine model using the simplifications imposed by the 1-D modelling approach is to establish the in-cylinder conditions in the model environment. Of particular importance is developing the heat transfer coefficient. With this established, parameters such as pumping work and volumetric efficiency can be compared with the experimental results. For this step, the software’s closed-cycle analysis tool was applied to the six calibration operating points introduced in Figure 1. Heat transfer was evaluated based on the experimentally measured cylinder pressure, with the Woschni correlation used to calculate the in-cylinder heat transfer coefficient in the model. This resulted in a single convective heat transfer coefficient multiplier of unity over the engine map using the Woschni model.

#### Phenomenological combustion model calibration

There are two distinct injection events in HPDI combustion: diesel and NG. To simulate this combustion process, the phenomenological DI-Pulse combustion model can be applied with separate fuels for the two injections; however, only a single set of calibration parameters can be used. Due to the small quantity of fuel injected in the pilot, only the ignition delay parameter is critical for the first diesel injection. For the main fuel injection – the natural gas – ignition is initiated from the heat release from the pilot diesel. Once the diesel has ignited, the in-cylinder temperature is such that the natural gas ignition delay is short and insensitive to the ignition delay multiplier. The vast majority of the energy released from the combustion (>95% in most cases) is from the natural gas combustion. As a result, the model calibration parameters relating to air entrainment, premixed combustion rate and diffusion combustion rate can be defined for the main NG injection and combustion without significantly impacting the diesel pilot. Two injectors were added for each cylinder to accommodate the HPDI combustion in the software, and the restrictions limiting the DI-Pulse model to a single injector were overridden^1^. At each calibration point, the injection quantity, duration (pulse width) and start of injection (SOI) from the experimental data set were used as inputs to the model. Defining injection quantity and duration with a fixed injector geometry indirectly defines the fuel density, which is a function of rail pressure for a compressible gaseous fuel. The injector opening and closing times are assumed to be very fast, resulting in an essentially rectangular injection profile.

The calibration process was carried out by extracting the heat release rate profile from the experimental data and then using the simulation software’s heat-released-based calculation and GA optimization for a single combustion cylinder. GA is an evolutionary multi-objective optimization method that was developed by Deb and Jain^35^ and is widely used to solve optimization problems with different levels of complexity. GA was employed to minimize the error of the model’s heat release rate compared to the experimentally measured pressure trace. During the optimization, the four parameters of the phenomenological model were varied from 0 to 2 for all of the selected calibration points of Figure 1 to reach an optimal value satisfying the target. Finally, as shown in Table 2, a single set of parameters was obtained from the calibration process, which can be used for all areas of the engine map (the fit of the model over the range of operating conditions evaluated is shown in the results section). Maintaining constant parameters across the engine map makes it possible to use the model as a predictive tool within the operating range covered by the calibration conditions.

**Table 2.** Phenomenological combustion model parameters and the obtained final values from the combustion calibration process.

Parameter	Entrainment rate	Ignition delay	Premixed combustion rate	Diffusion combustion rate
Value	0.67	1.68	0.07	0.36

A limitation of the phenomenological combustion model was the lack of precise injector opening and closing rates, especially for the NG injection. The dynamics of the HPDI injector result in injector needle performance that is sensitive to both NG and diesel rail pressures and the in-cylinder pressure. To assess model sensitivity, the injection rate profiles at three of the operating points were measured experimentally, and then the combustion model calibration was replicated for those points. The results showed no significant impact on the model’s predictive capability. Given that rate shapes were not available for the rest of the map and their prediction would introduce additional uncertainty, the fixed (rectangular) rate shape was used in the final model.

#### Full engine model (six-cylinder engine)

The obtained calibration parameters were incorporated into a 1-D thermo-mechanical engine system model. The full-engine model includes the cranktrain, all six cylinders with valves and ports, intake and exhaust systems, a charge-air intercooler and a turbocharger model. The model did not include an after-treatment system. Key component dimensions were provided by the project industry partner based on measurements taken from the engine or from component suppliers.

The multi-cylinder model can be used to simulate both steady state and transient operation. First, the engine speed is defined as either a fixed value (steady state) or a time series for transient modelling. The output (shaft) torque is defined as a target to be met. A simplified control strategy was utilized to model the engine’s transient operation. The engine model included a proportional-integral-derivative (PID) controller, which was used to get the transient cycle load requirement and modify the amount of NG injection in the injector to meet the torque target. A map-based approach (2-D look-up table based on defined speed and target engine torque) was used to define the injection parameters, including the SOI and injection duration (pulse width) for both NG and diesel fuels. The 2-D tables were populated using the experimental injection parameters for the steady-state points shown in Figure 1. The controller then interpolated between the defined points to define the injection parameters for a specific load and torque condition. This approach is compatible with what happens in the real injection system during the transient cycle, where combustion timing and rail pressure are adjusted based on engine speed and demanded torque. The procedure applies to both steady-state and transient modelling. It should be noted that the PID tuning parameters were constant for different transient profiles ranging from simple torque changes to more complex transient cycles such as WHTC.

#### NOx emission model development

During the combustion, unburned fuel and air move to the burned zone, leading to further temperature and composition changes. Then, NOx is calculated independently in each subzone, considering the air-fuel ratio and temperature. The version of GT-SUITE’s DI-Pulse combustion model used here includes a NOx prediction capability based on the extended Zeldovich mechanism (equations (1)–(3)). Although the model does not capture spatial variations in local conditions across the chamber, it uses the simulated reaction zone temperatures, composition (air-fuel ratio), trapped cylinder mass and combustion rate as inputs to the NOx model. Six calibration parameters are available to fit the NOx model to the experimental data.

(1) $$O+N_{2}⇆\mathrm{NO}+N\left(N_{2}\;\mathrm{oxidation}\;\mathrm{rate}\;\mathrm{equation}\right)$$

(2) $$N+O_{2}⇆\mathrm{NO}+O\left(N\;\mathrm{oxidation}\;\mathrm{rate}\;\mathrm{equation}\right)$$

(3) N+OH⇆NO+H(OHreductionrateequation)

The NOx model includes six different parameters, as shown in Table 3, which were calibrated using the GA optimization algorithm with the exact engine operating points used in the combustion model calibration. The NOx calibration multiplier adjusts the net rate of NOx formation (total NO formation rate minus the NO dissociation rate). The remaining multipliers were used to change the reaction rates in the Zeldovich mechanism by tuning the Arrhenius reaction rate coefficients and exponents as shown separately for each of the abovementioned reactions in equations (4)–(6).

**Table 3.** NOx model’s multipliers and the obtained final values from the NOx calibration process.

Multipliers	NOx calibration	N_2_ oxidation rate (F_1_)	N_2_ oxidation activation energy (A_1_)	N oxidation rate (F_2_)	N oxidation activation energy (A_2_)	OH reduction rate (F_3_)
Value	0.61	1.57	0.46	1.59	0.05	1.00

Once the engine combustion was calibrated, the NOx model optimization could be performed in the full engine model. The aim was to derive a single set of parameters for the NOx emission model, as shown in Table 3, which could also be used for other engine operating points.

The corresponding reaction rates of the Zeldovich mechanism and the application of NOx model calibration multipliers are shown in equations (4)–(6), where K_1_ to K_3_ are reaction rates, and T_b_ is the burned zone temperature:

(4) $$K_{1}=\;F_{1}\times 7.6\times 10^{10}\times e^{-38000\times A_{1}/T_{b}}$$

(5) $$K_{2}=\;F_{2}\times 6.4\times 10^{6}\times T_{b}\times e^{-3150\times A_{2}/T_{b}}$$

(6) $$K_{3}=\;F_{3}\times 4.1\times 10^{10}$$

### Methane emission model development

For natural gas engines, unburned methane emissions are a particular challenge as they have a global warming potential (GWP) of approximately 29.8 over a 100-year horizon^36^ and are hard to oxidize at typical exhaust stream temperatures. Any emissions of unburned fuel also reduce fuel economy and are restricted either as a GHG (US EPA) or as a pollutant with a fixed drive-cycle limit (Euro V and beyond). As a result, predicting CH_4_ is important when investigating engine and vehicle system configurations.

#### Phenomenological combustion model

The predominantly non-premixed nature of HPDI’s combustion process results in a methane emission profile that is substantially different from that of premixed NG engines. CH_4_ concentrations in the crevices, top land, and blowby gases are very low, and the mixing-driven combustion rate of the main gas jet makes it less sensitive to slow flame speeds under globally lean conditions. There are multiple potential sources of unburned methane in the direct injection NG combustion process. The most important ones under most conditions are thought to be i) overmixing when the NG penetrates past the pilot combustion products and partially premixes prior to reacting and ii) low momentum leading to poor mixing and slow reaction of NG at the end of the injection event due to the increased in-cylinder pressures and the loss of jet momentum as the injection event ends.^13^ Other reasons include the fuel in the injector sac and nozzle holes, which gradually enters the chamber, fuel jet impingement on the cooler surface and local extinction due to high turbulence creating holes in the flame front that unreacted fuel may enter the oxidizer. To predict the CH_4_ emissions, the 1-D model includes an over-mixing multiplier (CH_4_ increases by increasing this parameter due to over-mixing) and a partial oxidation rate multiplier (CH_4_ decreases by increasing this parameter). The aim was to calibrate those multipliers such that the predicted emissions match the experimental data. The results in the next sections show that the 1-D model overpredicted CH_4_ values, indicating that the 1-D model currently cannot take into account the specific mechanisms in the HPDI CH_4_ emissions.

#### Machine learning model development for exhaust emissions

1-D models lack spatial resolution for temperature or concentration fields and can struggle to accurately predict emissions of partial combustion byproducts and unburned fuel, particularly in non-premixed combustion. Although they lack representation of the fundamental physical processes, ML models can enhance the accuracy of these 1-D models. To enhance NOx emission predictions, given that the 1-D model is unreliable for CH_4_ predictions, an ML model was developed for this purpose, selecting five key inputs: engine speed, load, air flow, fuel flow, and exhaust gas temperature. These features were chosen by performing sensitivity analysis on the available experimental input data and because of either their significant influence on the NOx and CH_4_ emissions or because they are representative of other factors. For example, the engine load, air and fuel flow rate (impacting on equivalence ratio) can directly affect the emissions, while exhaust gas temperature in lack of in-cylinder temperature is a surrogate to represent the in-cylinder effects. Another reason for selecting these parameters is that the 1-D model reliably predicts them with 95% accuracy (see results section). These are the validated input data, enabling the ML model to use these data points from various driving cycles rather than relying on experimental tests.

**Figure 4.**
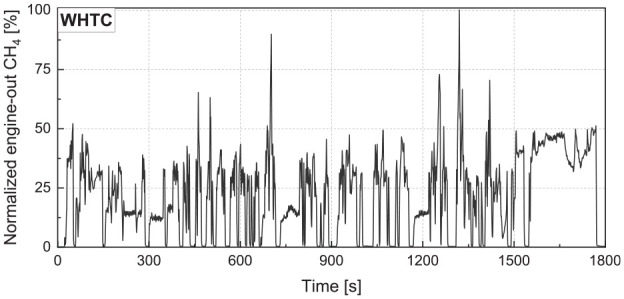
Engine-out CH_4_ mass emissions over the WHTC cycle, normalized by the maximum recorded emissions rate.

The ML model was trained and tested using the NG HPDI engine data from both the WHTC and WHSC transient tests. Using the data from such transient cycles, as shown for the WHTC in Figure 4, ensures that the ML model captures the cycle dynamics and the transient emission behaviour. Unlike steady-state engine operation, WHTC and WHSC have several load changes; feeding the data from these cycles as the train/validation set into the ML model ensures that the HPDI engine’s emission behaviour during tip-in and tip-out load changes are taken into account in the ML model. The data was cleaned and divided into training and testing sets using a random sampling technique. The data was scaled using standardization, which is less affected by outliers and is not limited to a certain range. Using stratified sampling, 10% of the cleaned data (275 data points) was allocated to the test set, and the remaining 90% (2475 data points) were used for training and validation.

The Random Forest (RF) model was utilized to forecast exhaust emissions. This model consists of an ensemble of decision trees created through the bagging technique, which involves random sampling with replacement. The decision tree and bagging classifier hyperparameters are combined in the RF model. It is based on a Regression Tree (RT) that repeatedly divides the data into branches using the Classification and Regression Trees (CART) algorithm. The following is the cost function that CART uses:

(7) $$J\;\left(\theta \right)=\frac{n_{\mathrm{left}}}{n}\mathrm{MS}E_{\mathrm{left}}\;+\frac{n_{\mathrm{right}}}{n}\mathrm{MS}E_{\mathrm{right}}\;$$

Where,

(8) $$\mathrm{MSE}\;\left(\theta \right)=\frac{1}{n}\sum_{i=1}^{n}{\left(y_{i}-{\hat{y}}_{i}\right)}^{2}$$

The left and right branches of the tree are also denoted by $n_{\mathrm{left}}$ and $n_{\mathrm{right}}$ . A well-regularized model is obtained by establishing a minimal threshold for the number of samples at each leaf node. Another important hyperparameter is the tree’s maximum depth.^37^

**Figure 5.**
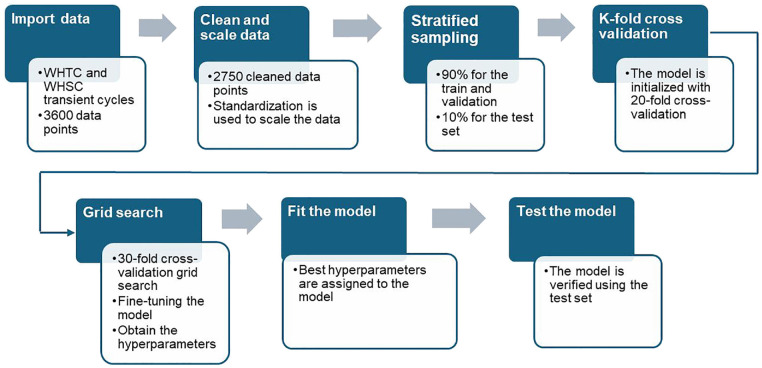
The process for training the RF model.

The workflow of model train and test processes is summarized in Figure 5. To initialize the model, K-fold cross-validation is implemented. The K-fold algorithm chooses one group to use as a fold in each iteration, trains a model on the remaining groups, and then assesses the model using the fold set.^38^

The model’s hyperparameters were fine-tuned by conducting a 30-fold cross-validation grid search, as illustrated in Table 4. The model was then adjusted using the determined hyperparameters. The training dataset was then used to fit the model. In order to find the optimal hyperparameter set, the grid search approach aims for the least error by testing every possible hyperparameter combination within a given range. The test set was then used to assess the model’s performance.

**Table 4.** ML model’s hyperparameters and the range used in the grid search method.

Model	Hyperparameters	Range used in grid search	Best hyperparameter
RF	n_estimator_ max_features	n_estimator_ = 3, 10, 30, 100 max_features = 1, 2, 3, 4	n_estimator_ = 100 max_features = 4

#### Transient model integration with machine learning models

One of the main benefits of the transient 1-D engine system model is its ability to predict engine behaviour over a range of different duty cycles that can represent different uses of a given vehicle or different powertrain architectures. The key parameters that can be reliably predicted include intake manifold and in-cylinder pressure, exhaust pressure and temperature, and air and fuel flow rates. It also provides a prediction of in-cylinder bulk temperatures (burned and unburned zone temperatures) and conditions that can be used in physics-based models to predict pollutant emissions but without the spatial resolution needed to resolve all the complex features of pollutant formation in the engine. Although pollutant formation depends on the local temperatures, the 1-D model does not include spatial resolution and needs tuning parameters in the emission model calibration process. Despite this limitation, the 1-D model uses in-cylinder burned zone temperature, which is indicative of in-cylinder conditions and has been shown to have strong correlations with emissions.^39,40^

**Figure 6.**
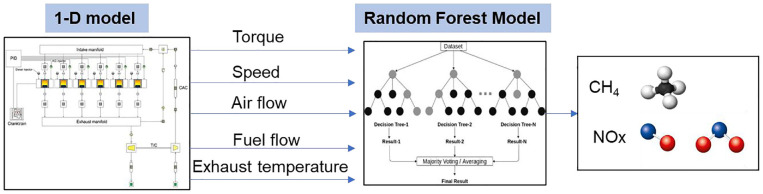
The validated 1-D transient model providing input features for the ML model over the transient cycle to predict CH_4_ and NOx emissions.

The 1-D model can also provide input real-time data for the powertrain controller or any ML-based model. For engine operation within the range of the validated data sets, a properly trained ML model that is supplied with relevant input parameters from the 1-D model may be able to predict emissions over a transient cycle with sufficient accuracy. In this paper, the opportunity of the transient model integrated with ML models is demonstrated to be used to predict exhaust emissions over transient cycles. The transient model can be integrated with the ML model, as shown in Figure 6, and its predictions, instead of those obtained from experimental data, are fed into the ML model.

## Results and discussion

The development and validation of the 1-D engine system model under steady-state and transient operation offers the potential to predict engine performance over a range of engine duty cycles. As the 1-D model generated critical parameters that are used as input features in the ML model, accurate prediction of fuel consumption, air flow, and exhaust gas temperature is essential. As mentioned previously, due to limitations in measuring the in-cylinder temperature in the test, exhaust gas temperature is selected as a surrogate to represent the in-cylinder effects and was identified as an impactful parameter during sensitivity analysis for the ML model. In addition, exhaust gas temperature is a critical hardware limit, impacting the turbocharger’s response. Predicting accurate brake-specific fuel consumption (BSFC) is also important in evaluating the impact of different engine or powertrain technologies on fuel economy using the transient model. Finally, accurate prediction of peak cylinder pressure (PCP) can help assess mechanical hardware and noise, vibration, and harshness (NVH) impacts.

### Combustion model validation

**Figure 7.**
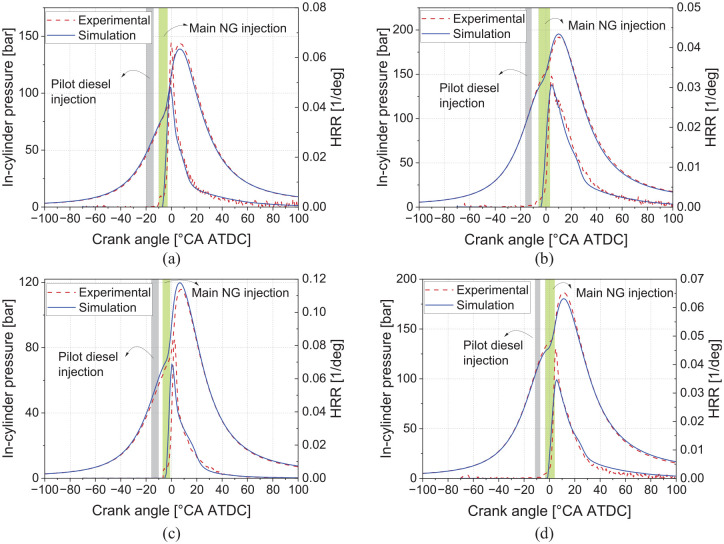
Comparison of the in-cylinder pressure and normalized heat release rate from simulation and experiment in (a) A50 (1200 RPM, 50% load), (b) B100 (1400 RPM, full load), (c) L50 (1000 RPM, 50% load), and (d) L100 (1000 RPM, full load) operating points.

Combustion parameters such as in-cylinder pressure, PCP, heat release rate, crank angle by which 50% fuel is burnt, and 10%–90% burn duration are important indicators of the combustion and, therefore, need to be verified in the model. The in-cylinder pressure trace and heat release rate (HRR) profile are compared between the experimental test and the 1-D model both for calibration (A50 and B100) and validation (L50 and L100) operating points (see Figure 1), which are shown in Figure 7. A minor difference in in-cylinder pressure and HRR is displayed by the 1-D model; this divergence is not expected to have a significant effect on other combustion model predictions, such as BSFC, PCP, and exhaust gas temperature.

**Figure 8.**
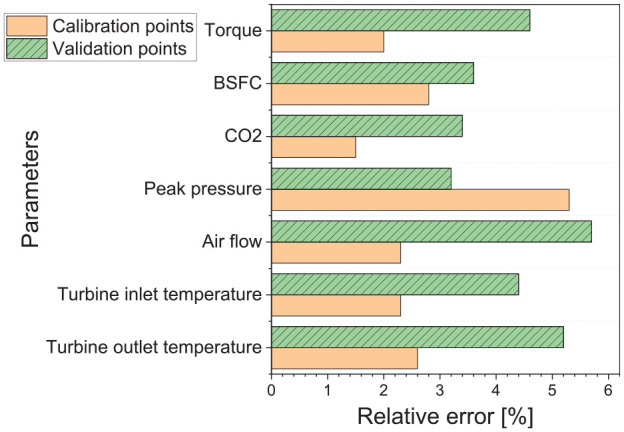
The relative error in steady-state calibration and validation points (Figure 1).

The model generally showed good performance at predicting key engine parameters, including fuel consumption, peak cylinder pressure, flow rates, and exhaust gas temperature. As would be expected, the calibration points showed higher accuracy than the verification points. However, the average (unweighted) percentage error across both calibration and validation points was small, as shown in Figure 8. All operating points use a fixed set of tuning factors (not optimized for each point), but they still show good accuracy both in calibration and validation points.

### Emission model validation

**Figure 9.**
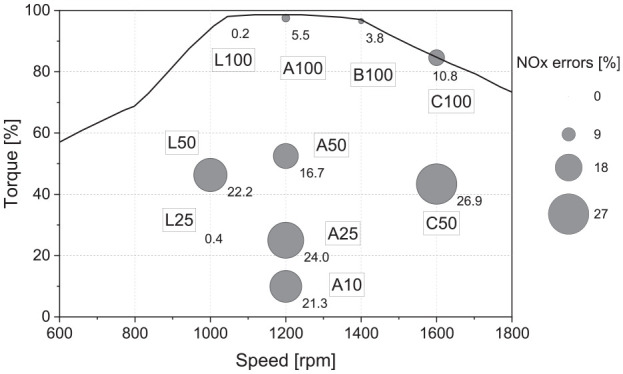
Distribution of simulation relative errors in the engine map for engine-out NOx emissions. Note that the *y*-axis is normalized regarding the maximum value.

The 1-D combustion model also had a reasonable ability to predict engine-out NOx emissions, as illustrated in Figure 9 for all the steady-state points evaluated. In general, NOx emission errors were substantially lower at higher loads. Although the airflow and exhaust gas temperature values in the 1-D model are acceptably close to those obtained from the experimental test, the NOx errors grow in the medium and lower load ranges. Other limitations can be related to the limited number of temperature zones in the model and corresponding uncertainties on both post-reaction zone temperature and mixing rates that are critical to accurate NOx prediction. Although some of the single points in the NOx model might have a high relative error, the results in the next sections show that the cumulative errors in transient cycles are less than 10%.

The 1-D combustion model’s ability to predict unburned fuel (CH_4_) emissions depends on the multi-zone conditions and the two calibration parameters that can, at best, only partially capture the complexity of CH_4_ emissions from an HPDI engine. With a single pair of tuning parameters, the model tends to overestimate the CH_4_ in the NG HPDI engine. The estimated CH_4_ emissions from the simulation model are 1.5 to 5 times higher than the experimental values in lower loads, while in higher loads, the model overestimates the experimental data by a factor of 12 to 22. Variations of this magnitude indicate that the 1-D model, as configured, will not provide reliable predictions of CH_4_ emissions in the NG HPDI engine. While the 1-D model is based on physical processes, the sensitivity of CH_4_ emissions to the injection and combustion of an underexpanded premixed gaseous fuel jet introduces more complexities than the current model can resolve. For this reason, alternative methods, such as verified machine learning models, need to be implemented to accurately predict CH_4_ emissions over different drive cycles.

### ML for emission prediction

**Figure 10.**
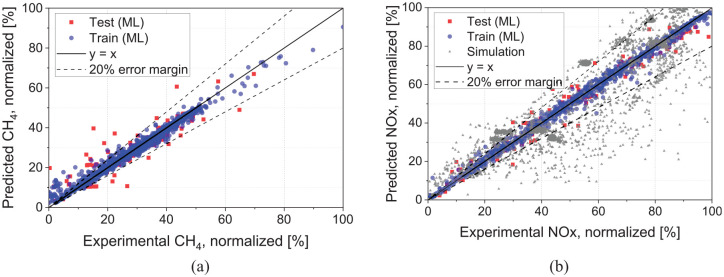
(a) ML model’s predictions for CH_4_ emissions in the train and test data sets and (b) comparison of ML model’s predictions with 1-D simulation results for NOx emissions.

As an alternative to a physics-based model, a data-driven ML model can provide estimation based on correlations within an extensive set of training data. Within these constraints, the ML model can provide accurate results for CH_4_ and NOx emissions, as shown in Figure 10. Due to the large number of data sets required to train the ML model, the comparison is conducted for all the data points from the cycle-based (WHSC and WHTC) emissions runs rather than the steady state points. Figure 10(a) shows the distribution of the experimental CH_4_ data set against the ML predictions. Most data points are in ±20% error margin using the ML model. Moreover, NOx results obtained from the ML model tend to have lower deviation from the experimental results compared to the 1-D simulation results over the same transient cycles, as shown in Figure 10(b).

**Figure 11.**
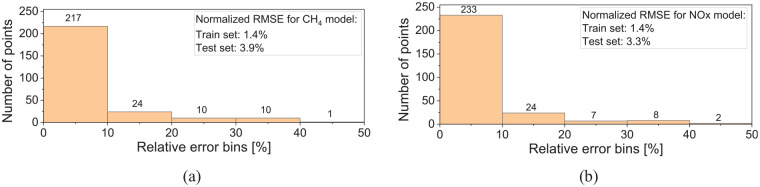
Error distribution of the ML model in the test data set for (a) CH_4_ and (b) NOx emissions.

Figure 11 illustrates the error distribution, which shows that for both NOx and CH_4_ emissions, 80% to 90% of the test set had less than 10% error. The relative error distribution shows that most predicted points have less than 10% error. The machine learning model’s root mean squared error (RMSE) is computed for both the training and test sets. The RMSE is calculated from the final ML model with the ideal hyperparameters and is scaled as a percentage of the maximum value of the label. The normalized RMSE (ratio of RMSE to maximum CH_4_ or NOx test value) is 2% and 4% for the train and test sets, indicating the ML model accuracy in estimating the emissions. Moreover, since the RMSE difference between the train and test sets is small, the overfitting does not occur in the trained models. Increasing the test set size to 25% of the data (75% for the training set) marginally increased the RMSE values. However, most of the data points (80%–90%) in the test set were within 10% of the experimental results for both NOx and CH_4_ prediction, similar to what is seen in Figure 11.

**Figure 12.**
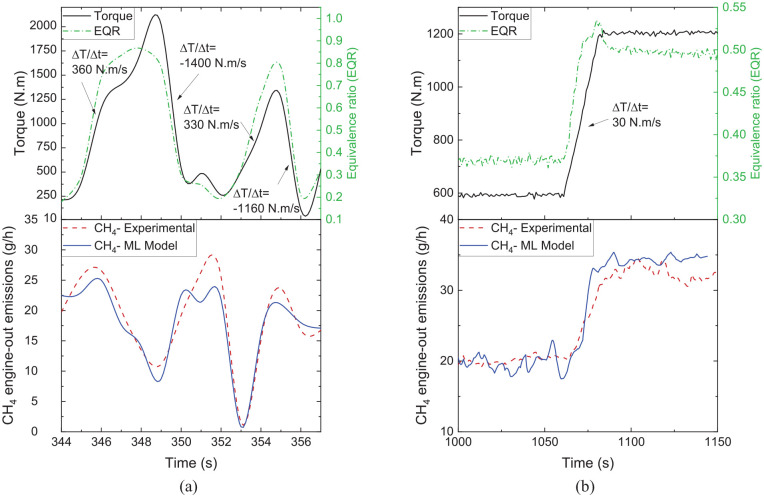
Transient load shifts, equivalence ratio (EQR) changes and the corresponding CH4 emissions (experimental and ML model) during segments of (a) WHTC cycle (average speed of 1000 rpm) and (b) WHSC cycle (600 to 1200 N.m at 1200 rpm).

Since the ML models are generated based on the transient data, they can capture the emission behaviour during load changes, as depicted in Figure 12 for CH_4_ emissions over segments of WHTC and WHSC cycles. As seen in Figure 12(a), the WHTC cycle had drastic load changes compared to the WHSC cycle, and the results showed a good agreement between the ML model and experimental data over both cycles in capturing the transient CH_4_ emissions.

### Engine transient operation

**Figure 13.**
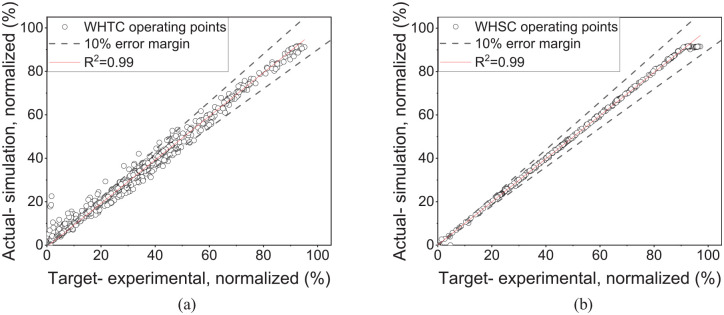
Comparison between experimental and simulation results for engine torque in (a) WHTC and (b) WHSC cycles.

A principal benefit of a 1-D engine model is to simulate engine performance and operating conditions over a wide range of transient duty cycles. As speed was used as an input parameter, the 1-D model’s ability to follow the desired duty cycle is best demonstrated by comparing the output torque vs. the torque demand, as well as turbocharger speed, air and fuel flow rates, exhaust gas temperature, and emissions for WHTC and WHSC standard transient cycles. Figure 13 shows the transient model’s capability to meet the torque response over these two transient cycles. As seen in Figure 13, there is a good agreement between the model’s predictions and experimental data, and the model can capture the transient torque during the cycles very well. Variations observed in the WHTC (Figure 13(a)) are a result of the response of the PID controller for the fuel injection quantity control. These are apparent because of the WHTC’s more rapid load and speed changes compared to the WHTC.

**Figure 14.**
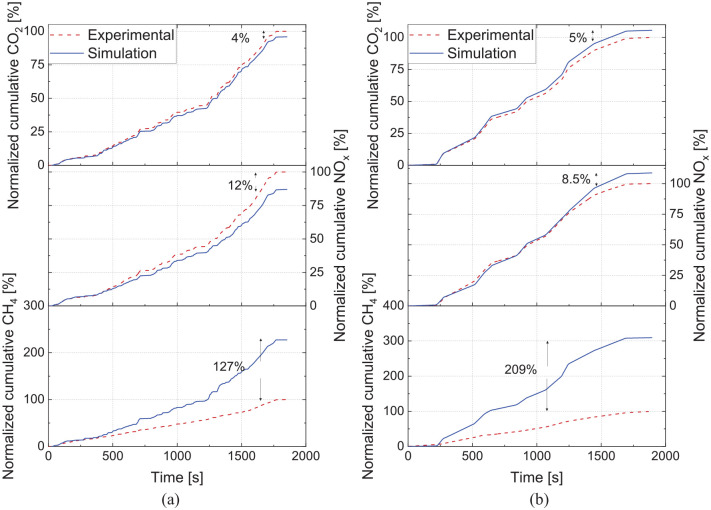
Comparison between experimental and simulation results for cumulative CO_2_, NOx and CH_4_ values for (a) WHTC and (b) WHSC cycles.

Alongside the model’s ability to follow the torque demand, the transient model accurately predicts CO_2_ and NOx transient emissions. As shown in Figure 14, the cumulative values show less than 12% error in engine-out NOx predictions and 5% error for CO_2_ values, highlighting the model’s capability to be used for system-level evaluations. However, the 1-D model cannot accurately predict the CH_4_ emissions, as shown in Figure 14, since it cannot capture the complex mechanisms for the CH_4_ emissions in the underexpanded NG jet in the HPDI engine. As a result, the ML model will be used alternatively to estimate the CH_4_ emissions over the studied drive cycles.

**Figure 15.**
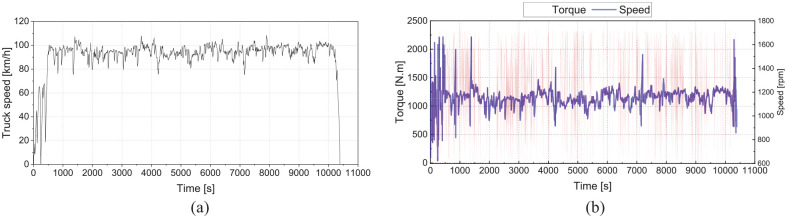
(a) The real-world truck testing speed profile over the Highyway 2 cycle, and (b) engine load and speed of the truck over the Highyway 2 cycle.

Along with accurately predicting engine performance, CO_2_ and NOx emissions, the 1-D engine model can provide input features for the ML model to predict NOx and CH_4_ emissions over transient engine duty cycles that are different from those used in the experimental testing. This capability is demonstrated over an experimental cycle. Figure 15(a) shows the vehicle speed and corresponding engine torque and speed for a Peterbilt Model 579 class 8 truck equipped with a Cummins X15 diesel engine travelling on Highway 2 (HYW2) from Calgary to Edmonton, Alberta, Canada – data collected by the University of Alberta.^10,41^ As the engine used in the Highway 2 study had a torque and speed profile similar to the modelled HPDI engine, it provides an interesting alternative duty cycle to assess the performance of the HPDI engine over a representative use case. The engine speed and torque profiles (Figure 15(b)) can be used as inputs into the 1-D model of the HPDI engine to simulate the engine’s operation throughout the cycle. It is interesting to note that although the truck is predominantly cruising on the highway, the engine torque profile has many intense transients (Figure 15(b)) resulting from varying road grades and traffic conditions. Since transient load changes in this cycle (±200 N.m/s) align with those on which the ML model is trained (WHTC and WHSC cycles), the ML model can be properly applied to this cycle to estimate the CH_4_ emissions.

**Figure 16.**
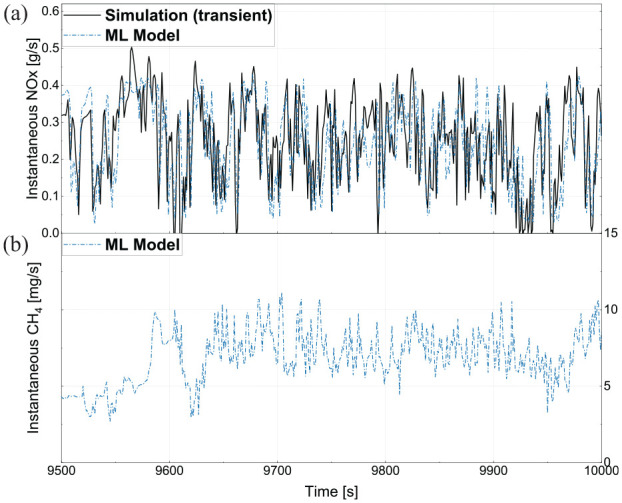
(a) The engine-out NOx emission results for a segment of Highway 2 cycle predicted by the 1-D model, and (b) the CH_4_ results predicted by the ML model with the input features from the transient 1-D model.

In addition to predicting engine performance and fuel consumption, the engine 1-D model was able to predict NOx emissions and provide input data to the ML model for both CH_4_ and NOx predictions. To investigate performance over the transient conditions of the model, time-resolved emissions predictions for a portion of the Highway 2 simulated duty cycle are shown in Figure 16. The variations of NOx emissions are attributed to engine load variations where in higher loads, NOx flow rate is also higher. Moreover, the 1-D model provided input features for the ML model so that the ML model could predict NOx and CH_4_ emissions. As seen in Figure 16(a), predicted NOx emissions from transient simulation and ML models are in good agreement. Moreover, the ML model predicted CH_4_ emissions, which can be seen for a portion of the cycle in Figure 16(b). The intense transients of the engine load over the Highway 2 cycle lead to variations in CH_4_ emissions, although levels remain relatively low, ranging from 5 to 10 mg/s (for comparison on an equivalent GHG basis, CO_2_ emissions were in the range of 10–40 g/s for the 500 s period shown in Figure 16).

**Figure 17.**
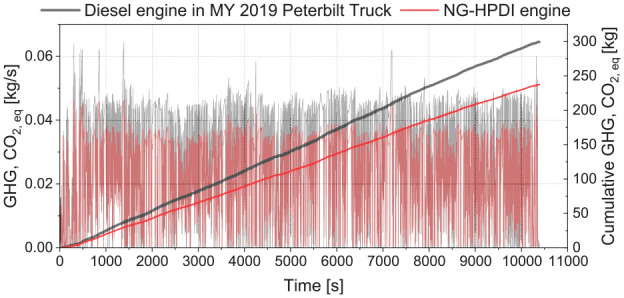
Comparison of GHG emissions from the diesel engine of the 2019 Peterbilt truck and the HPDI engine over Highway-2 drive cycle.

As the CH_4_ emissions are predicted over the drive cycle, by considering the global warming potential of CH_4_, the total GHG emissions as a CO_2_ equivalent can be calculated in the HPDI engine and compared to those collected from the truck’s diesel engine during the test. Figure 17 shows this comparison, indicating that the HPDI engine had a 21% lower GHG than the diesel engine over the Highway-2 drive cycle with 100% cargo capacity. These results only consider emissions of CO_2_ and CH_4_, neglecting other species (such as N_2_O, which is a small contributor to diesel engine GHGs^42–44^) or the behaviour of the aftertreatment system. For both HPDI and diesel engines, the change in CO_2_ across the aftertreatment is small, as CO and non-methane hydrocarbon engine-out emissions are low under typical heavy-duty cruising conditions, and current oxidation catalysts do not provide substantial conversion of CH_4_ under the lean exhaust conditions of the modelled HPDI NG engine.

**Figure 18.**
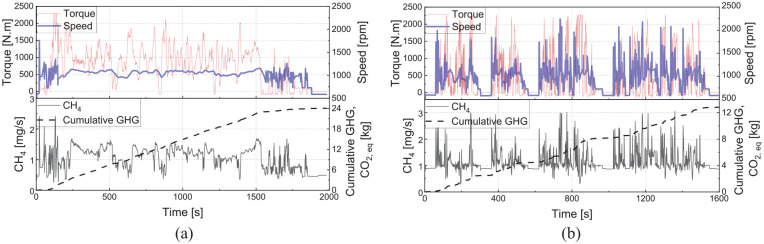
Engine speed and torque along with corresponding CH_4_ and cumulative GHG emissions for (a) HHDDT and (b) CSHVR cycles.

The integrated transient and ML model can be applied to any drive cycle, including regulatory ones, such as Heavy Heavy-Duty Diesel Truck (HHDDT) and City Suburban Heavy Vehicle Route (CSHVR). The integrated model was applied to these cycles, as shown in Figure 18, and the CH_4_ emissions were predicted along the cycle. The results indicated that the share of CH_4_ emissions in total GHG emissions over HHDDT and CSHVR were 0.2% and 0.5%, respectively. The higher CH_4_ share over CSHVR is due to more low-load operation. Interestingly, these are both lower than the CH_4_ share of 1% of total GHGs over the Highway-2 cycle. While the reasons for this are not certain, comparing the engine torque and speed profiles for the three duty cycles suggests that the Highway 2 cycle faced more frequent and rapid torque transients. This reinforces the importance of conducting evaluations over a wide range of engine duty cycles – a factor that becomes even more important when assessing the role of alternative powertrain systems, such as hybrid-electric, to reduce total GHG emissions from heavy-duty vehicles.

As summarized in Table 5, the developed transient model has accurate predictions for fuel consumption and NOx emissions when compared with the results of two experimental transient cycles, that is, WHSC and WHTC. The developed machine learning model could also accurately predict NOx and CH_4_ emissions, as shown in Figures 10 and 11. The cumulative errors of the ML NOx and CH_4_ models over WHSC and WHTC are very small, as shown in Table 5, mainly because the models are trained using these cycles. When comparing the ML model and 1-D NOx results, the cumulative differences are between 6% and 12%, highlighting that both models apply to predicting NOx emissions over transient cycles. However, given the large inaccuracy in the 1-D predictive model for CH_4_ emissions, it is not included in Table 5 for comparison. Finally, the ML model showed that the CH_4_ to total GHG emissions ratio is higher in the WHTC and WHSC cycles since these cycles have several low-load operations.

**Table 5.** Summary of the 1-D and ML model’s errors and differences.

Cycle	1-D Simulation cumulative FC error (%)	1-D Simulation cumulative NOx error (%)	ML cumulative NOx error (%)	Cumulative NOx differences (%)	Cumulative CH_4_ error (%)	CH_4_ (ML) to GHG ratio (%)
	$\left|\frac{FC_{1-D}-FC_{\mathrm{test}}}{FC_{\mathrm{test}}}\right|$	$\left|\frac{\mathrm{NO}x_{1-D}-\mathrm{NO}x_{\mathrm{test}}}{\mathrm{NO}x_{\mathrm{test}}}\right|$	$\left|\frac{\mathrm{NO}x_{\mathrm{ML}}-\mathrm{NO}x_{\mathrm{test}}}{\mathrm{NO}x_{\mathrm{test}}}\right|$	$\left|\frac{\mathrm{NO}x_{\mathrm{ML}}-\mathrm{NO}x_{1-D}}{\mathrm{NO}x_{1-D}}\right|$	$\left|\frac{\mathrm{CH}4_{\mathrm{ML}}-\mathrm{CH}4_{\mathrm{test}}}{\mathrm{CH}4_{\mathrm{test}}}\right|$	$\frac{CH_{4}}{\mathrm{GHG}}$
WHSC	4	12	1.5	9.4	1.8	1.15
WHTC	5.5	8.5	1.1	6.8	2.5	1.4
HYW-2	–	–	–	8	–	1
HHDDT	–	–	–	11.8	–	0.2
CSHVR	–	–	–	10.2	–	0.5

## Conclusion

This work describes the development and use of a 1-D engine system model to predict the performance of a 6-cylinder high-pressure direct injection (HPDI) of a natural gas engine in both steady-state and transient operations. The 1-D engine transient model is coupled with a machine learning (ML) model to predict unburned methane and NOx emissions. The 1-D engine model provides physically relevant parameters as inputs to the machine learning model, allowing the correlations developed in the ML model to predict emissions over a range of transient in-use and regulatory cycles. The main findings of this paper are:

- The developed steady-state 1-D model was built on an extensive dataset from engine test data and can be used to accurately predict the pilot diesel-main direct injection of natural gas combustion process over a wide range of operating conditions with a fixed set of tuning parameters. The model’s error is less than 5% in predicting main parameters, such as torque, BSFC, peak pressure, air flow and exhaust gas temperature.
- Based on test data from transient duty cycles, the 1-D model was demonstrated to follow the target torque profile and provide accurate predictions for fuel consumption and cumulative engine-out CO_2_ and NOx emissions. However, due to the complex sources of unburned methane (CH_4_) emissions in the HPDI engine and the lack of spatial resolution in the phenomenological model, the 1-D model could not predict the methane emissions correctly.
- For the present engine configuration, after validation based on test data, the output from the 1-D engine model can be used to provide physically relevant input features to a machine learning model tuned on transient duty cycles to predict emissions, including engine-out NOx and unburned methane with errors under 10% over the transient test cycles used in the model development. The combined method could accurately predict the transient CH_4_ emissions during transient engine load changes.
- Combining the predicted CO_2_ from the 1-D engine model and CH_4_ from the machine learning model provides a prediction of net greenhouse gas emissions over various engine duty cycles. The results from the model showed that the methane emission’s share in total greenhouse gas emissions for the natural gas engine ranges from 0.2% to 1.4%.
- The integrated model results demonstrated that the natural gas HPDI engine’s net greenhouse gas emissions can be evaluated and compared to an equivalent diesel engine over a real-world drive cycle. The model results indicate that the net greenhouse gas emissions are 21% lower over a high load highway drive cycle.

This study demonstrates the potential of a 1-D engine model built on extensive validation using test data for predictive purposes in evaluating steady and transient engine performance and emission behaviour over a wide variety of duty cycles. This paper has shown that the complex combustion process of an HPDI engine can be reasonably and accurately predicted using a 1-D phenomenological model with a fixed set of calibration parameters applicable to real-world drive cycles. In addition, since the 1-D model could not predict CH_4_ emissions correctly, the 1-D engine model was integrated with a machine learning model and substantially improved the estimation of CH_4_ emissions over various drive cycles. Having a reliable, fast-running engine model integrated with machine learning models that can predict transient operation with sufficient accuracy is essential for evaluating new engine and powertrain technologies over various duty cycles.

## Appendix

### Abbreviations

BMEP brake mean effective pressure

BSFC brake specific fuel consumption

BTE brake thermal efficiency

CART classification and regression trees

CLD chemiluminescence detector

CFD computational fluid dynamics

CR compression ratio

CSHVR City Suburban Heavy Vehicle Route

EQR equivalence ratio

FC fuel consumption

FID flame ionization detector

GA genetic algorithm

GHG greenhouse gas

GPR Gaussian process regression

GVWR gross vehicle weight rating

GWP global warming potential

HDV heavy-duty vehicle

HHDDT Heavy Heavy-Duty Diesel Truck

HPDI high-pressure direct injection

HRR heat release rate

ML machine learning

MLP multi-layer perceptron

NG natural gas

NMHC non-methane hydrocarbon

NVH noise, vibration, and harshness

OEM original equipment manufacture

PCP peak cylinder pressure

PID proportional–integral–derivative

RF random forest

RMSE root mean squared error

RT regression tree

SOI start of injection

SVR support vector regressor

THC total hydrocarbons

WFS Westport Fuel Systems

WHR waste heat recovery

WHSC world harmonized stationary cycle

WHTC world harmonized transient cycle

ZEV zero-tailpipe emission vehicle
